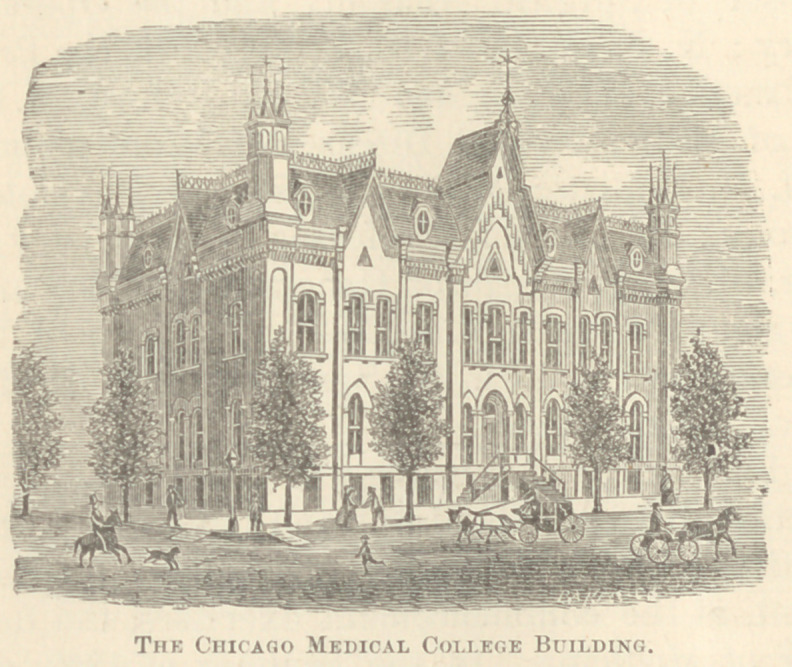# Early Medical Chicago

**Published:** 1876-03

**Authors:** James Nevins Hyde

**Affiliations:** Chicago


Daniel Brainard.

THE

### Chicago Medical Journal

AND

### EXAMINER.

Vol. XXXIII. — MARCH, 1876. — No. 3.

### Original Communications.

### EARLY MEDICAL CHICAGO.

By JAMES NEVINS HYDE, A.M., M.D., Chicago.

To assert to-day that the age of men and cities should be estimated, rather by the march of events than by the lapse of time, is to merely utter a truism. There are tapestries now hanging in the palaces of Venice, that have been undisturbed since the Venetian Dandolo carried the walls of Constantinople. How little of change has each succeeding half century wrought in the apartments which now display the faded furnishings of a long-departed Doge! And yet, in the purview of history, how venerable was the royal prophet of Israel in the Assyrian Court, who had exchanged the captivity of his childhood for the government of a province, and survived the rise and fall of three dynasties, when Cyrus entered the Babylonian capital by the bed of the Euphrates!

By the transit of time merely, Chicago may be counted as yet young, but she is really old in the measure of her experience. Dismissing for the moment the charge which is generally, and possibly justly, brought against her citizens, that they are prone to exaggerate the rapidity of her growth and the extent of her development, these are yet facts which challenge investigation. Here is a city of nearly half a million of inhabitants, where fifty years ago was a morass, untenanted and almost untenantable. The great concentration of human energies requisite to effect such a rapid metamorphosis, is difficult of realization. No better illustration of the rapidity of succession of events within this limited period can be found, than in the fact that an experience of the early days of Chicago has come to be regarded with much of the veneration that attaches to a remote antiquity. And yet the child who first saw the light in the infancy of the city, should to-day be only in the meridian of life.

I purpose to present a brief sketch of the pioneers in this field—the predecessors of the large body of medical men who are now engaged in the practice of their profession in this great metropolis. The paucity and imperfection of these details are largely due to the difficulty inseparable from their collection.

The early history of Chicago, and the first records of its medical men and practice, are intimately associated with its old fort. Even as early as the treaty of Greenville, O., which is dated August 3, 1795, there is some reference to a fort, built at the junction of the lake and the river.* This was, however, a small stockade erected for the protection of French traders, at the point where the north and south branches of the river unite, some remains of which were still to be seen in the year 1818.

Fort Chicago† was built by the United States Govern-

* Sketches of the Country on the Northern Route, from Belleville, Ill., to the city of New York, and back by the Ohio Valley, with a sketch of the Crystal Palace. Jno. Reynolds. Belleville. 1854.

† In the papers of Mr. J. H. Kinzie, and according to the statement of Mrs. Gen. Whistler, lately in Chicago, it appears that this fort was called by the name of Gen. Dearborn as well as its successor. Mr. Kinzie's papers were destroyed in the Great Fire, which consumed the library of the Chicago Historical Society.

ment in 1804, and but little is known of it except that it was provided with a subterranean passage and sally-port, extending from the parade ground to the river.* The Indian name, which it bequeathed to the city, is variously interpreted as referring to the wild onion, or the pole-cat; but the natives themselves asserted that it was the title of an Indian chief who had been drowned in the river. In the manuscript letter of M. de Ligney at Green Bay to M. de Siette among the Illinois, dated in 1726, the name is spelled, "Chicagoux."†

The narrative of the massacre at Fort Chicago by the Indians, in 1812, has been detailed in such fullness, that it can not find a place here. It is now a matter of historical record. The account given by Mrs. Helm, however, in the very readable volume of Mrs. Kinzie,‡ is interesting in this connection, as it relates in part to the surgeon of the fort—Dr. Isaac V. Van Voorhees.§

It appears from Mrs. Helm's narrative, that Dr. Van Voorhees came up to her during the very hottest part of the engagement. He was severely wounded, having received a ball in the leg, and his horse had also been shot under him. Every muscle of his face was quivering with agony. Some conversation ensued between the two, when, writes Mrs. Helm, "a young Indian raised his tomahawk at me. By springing aside, I avoided the blow which

* The fort was then occupied by fifty men and armed with three pieces of artillery, transported thither on the U. S. Schooner Tracy, Dorr, master. This vessel did not cross the bar and enter the river, but anchored half a mile from the shore, and discharged its freight by boats, attracting the presence of some 2,000 Indians, who came to view the "big canoe with wings." (See Chicago and its Suburbs, by Everett Chamberlin. Chicago. 1874. Also, Chicago Antiquities, No. 2, by H. H. Hurlbut, Esq. Chicago. 1875.)

† The name is also spelled by various authorities, Chikajo, Checagua, and Chekagua. (See Frauquelin's map, 1684.)

‡ "Wau-Bun;" or the Early Day in the Northwest. By Mrs. Jno. H. Kinzie. New York and Chicago. 1857.

§ His name is also given Voorhees and Voorhes. See "My Own Times." By Jno. Reynolds, Ill. 1855. Also, "Annals of the West." J. R. Albach. Pittsburgh. 1857.

was intended for my skull, but which alighted on my shoulder. I seized him around the neck, and while exerting my utmost strength to get possession of his scalping-knife, which hung from a scabbard over his breast, I was dragged from his grasp by another and an older Indian. The latter bore me, struggling and resisting, to the lake. Notwithstanding the rapidity with which I was hurried along, I recognized, as I passed them, the lifeless remains of the unfortunate surgeon. Some murderous tomahawk had stretched him upon the very spot where I had last seen him."

I have purposely omitted the conversation which is reported to have occurred between the two, and which is exactly repeated in almost every account of the massacre, since it reflects but little credit upon the wounded officer. It represents him as in an agony of terror, and his companion as reproaching him for his pusillanimity. But there are several circumstances which the professional reader can not fail to consider, before consigning the name and reputation of Dr. Van Voorhees to historical obloquy. Without questioning the veracity of the writer, it is evident that the incidents narrated rest upon the recollection of a single individual, and that individual a woman surrounded by circumstances of extreme peril and excitement. She appears as the heroine of the story, and on that account due allowance should be made for partiality of statement. Dr. Van Voorhees, moreover, was evidently suffering from his wounds. We only learn of that inflicted upon the leg. What other injuries he may have sustained—whether of the brain, chest or abdomen—we can not know. Whether, indeed, he was wounded even unto death, and sank lifeless to the ground soon after, rather as the result of this than from the blow of a tomahawk, can not be determined. Jurists, as well as medical men, learn to accept with great reserve statements made either *in articulo mortis* or in the immediate peril of violent death. Too many surgeons have exhibited not only a consummate skill, but a splendid courage upon the field of battle, for their professional brethren to doubt the compatibility of these virtues. They will only remember, therefore, of their martyred representative in the battle of Chicago, that he was sorely wounded in the discharge of his professional duties, and that he died the death of a soldier.*

After the encounter, the survivors must have sadly missed the attentions of the dead surgeon. Mr. Kinzie soon applied to an old Indian chief, who was reputed to possess some skill in these matters, to extract a ball from the arm of Mrs. Heald—the wife of the captain who had commanded the fort. "No, father," was the response, "I can not do it, it makes me sick here"—said the Indian, pointing to his heart. Mr. Kinzie then performed the operation himself with his penknife. The accoutrements of the surgical department had meantime fallen into possession of the Indians. Later, we learn that a French trader, a M. du Pin, was in the habit of supplying medicines as well as medical advice to those in need of either; and, on one occasion, we hear of his prescribing for the infant of a Mrs. Lee, who was one of the captives. It appears that his efforts were not unattended with success.

In the year 1816, the fort was rebuilt by the Government, under the supervision of Captain Hezekiah Bradley, who is reported to have been so zealous in the discharge of his duties, that he enlisted officers as well as soldiers in the prosecution of the work, and even had wooden pins fashioned, in order to fasten together the timbers of the buildings, and thus economize his supply of spikes and nails. At this time, also, the entire tract of land was ceded to the United States by the Pottawatomies. With them, according to Judge Caton,† Chicago had

* In the official account of the engagement, the loss of Dr. Van Voorhis (for so his name is given by Captain Heald) is deeply deplored, and nothing is said that reflects in the slightest degree upon his character as an officer and surgeon.

† "The last of the Illinois, and a Sketch of the Pottawatomies." By John Dean Caton, LL.D. Chicago. 1870.

ever been a favorite resort. Here, they had chosen to hold their great councils, and here, they concluded both the first and last treaty with our Government.

In the year 1818, the place was visited by Mr. Gurdon Saltonstall Hubbard, who is now a resident of Chicago and the oldest representative of its early days. At that time, besides the fort, there were but two residences standing, one that of Mr. John Kinzie, the other of Antoine Houlmette. It may be mentioned here that Mr. Hubbard, at a later period, (1834), erected the first brick building ever reared in Chicago.*

Two years later, we find recorded the name of another medical gentleman, Dr. Alexander Wolcott, of Connecticut. He was born on the 14th day of February, 1790, at Windsor, Ct., and was the son of Alexander Wolcott, Sr., and Frances Burbank. His father was, with the writer of these pages, a descendant of William Hyde, of Hartford, Ct. (1636), and was graduated at Yale College, becoming afterward a distinguished lawyer and Justice of the Peace in Windsor. He subsequently removed to Middletown, Ct., where he was appointed collector of the customs and member of the constitutional convention of 1818. President Madison subsequently nominated him as a Justice of the Supreme Court of the United States, but the federalists in the Senate succeeded in preventing the appointment.† The distinguished Governor, Henry Wolcott, was his near relative.‡

Dr. Wolcott was graduated at Yale College in 1809,§ and must have received his degree in medicine elsewhere, as the medical department of that University was not established until 1814. He came to Chicago in 1820 as an Indian agent of the Government, succeeding to the

* This building stood on the corner of South Water and La Salle streets, and was for some time known as "Hubbard's Folly."

† Genealogy of the Hyde Family, by Chancellor Reuben Hyde Walworth, LL.D., Albany, N. Y. 1864. Vol. 2, p. 1121.

‡ History of Connecticut, by G. H. Hollister. New Haven. 1855.

§ Catalogus Collegii Yalensis in Novo-Portu in Republica Connecticutensi. MDCCCLXV.

position of Mr. Charles Jewett, and was soon after married to Miss Ellen Marion Kinzie, then sixteen years old, by John Hamlin, a Justice of the Peace, summoned to the village in order to perform the ceremony. The young lady was the daughter of John H. Kinzie, Esq., and was born in Chicago in the month of December, 1804, being indubitably the first child of white parents born on the soil. Dr. Wolcott died in 1830, and his widow was united in a second marriage to the Hon. Geo. C. Bates, of Salt Lake City. Through the kindness of Henry H. Hurlbut, Esq., of Chicago, I am enabled to present this *fac simile* of the lady's autograph:

By a stupid act of our local legislators the name of Wolcott street, which served as an historical landmark of this early resident, was changed to North State street.

I am informed by the Hon. John Wentworth of this city, in a recent letter, that Dr. Wolcott during his lifetime served in the capacity of an army surgeon. It seems, however, tolerably clear that he performed the duties first named, residing as he did outside of the fort; though it may well be believed that there must have been a demand for his professional services such as he could not but gratify, and indeed his selection for such a post must have resulted in part from his attainments as a physician.

The outside world must have known but little of the infant settlement in 1823. For in a Gazetteer * published at that date, the information respecting Chicago is extracted from an account given in "Schoolcraft's Travels." It appears that some twelve or fifteen houses had been erected, which were occupied by some sixty or seventy inhabitants. "The country around is the most fertile and beautiful that can be imagined. It consists

* Gazetteer of the States of Illinois and Mississippi, by Lewis C. Beck. 1823.

of an intermixture of woods and prairies, diversified with gentle slopes sometimes attaining the elevation of hills (!), irrigated with a number of clear streams and rivers, which throw their waters partly into Lake Michigan and partly into the Mississippi river. It is already the seat of several flourishing plantations."

During the year 1822, there were eighty-seven men in the garrison and one death occurred; during the ensuing year, there were ninety-five men, and of these, three died. The fort was then abandoned, but occupied again in 1828, one year after the passage of a bill in the legislature for the construction of the Illinois and Michigan Canal. This was the genial warmth that hastened the germination of the seed destined to produce so worthy a harvest. Game was abundant, the land was fertile, and corn easily grown. Occasionally the mail was brought from Peoria on horseback. But Chicago was yet unborn.

It must be admitted that the infant first opened its eyes upon Lake Michigan, in an uneventful period of history. No great war was in progress, and commonplace men were in power. William IV., plainest and homeliest of royal blood, was seated on the British throne, and co-operating with the whig party in reforming parliamentary representation, and in restricting the operation of the oppressive corn laws. During the Revolutionary War he had figured in the dance, at No. 1 Broadway, with the loyalist belles of New York City. The triumph of the constitutional party in France had made a king of Louis Philippe—a man as incapable of exciting the affections of others, as he was destitute of magnanimity himself. He still preserved the recollection of his wandering tour in America. General La Fayette, now seventy years old, had returned to France, rewarded with the friendship of Washington and the gratitude of the United States. Otho I. had just been bolstered up on the throne of Greece. Poland had sunk down disarmed—the helpless victim of the iron sceptre of the Muscovite. Then, as now, a Don Carlos at the head of a faction of Carlists, was agitating Spain. Perhaps the only man in Europe, who was making himself felt as a power, was Daniel O'Connell, who was threatening the repeal of the National Union in Parliament at the head of a legion of Irishmen.

It seemed as though the succession of splendid events, that had culminated at Waterloo, and even lighted up by reflection the gloom of St. Helena, had been followed by a general reaction, in which all the great States participated.

In our own country also, the hero of the battle of New Orleans had laid aside his sword in order to discharge the more peaceful duties of the chief magistracy. The population of the country, according to its then recently taken census, amounted to twelve and one-half millions, a figure three times greater than that obtained by the first colonial census, and yet but one-fourth of that which should represent the people of the United States in 1870. It was the semi-centennial decade of our first hundred years of national life. Already the sentiments and passions, that were later to culminate in civil war, had been expressed in the halls of Congress. The great speeches of Webster and Hayne had been delivered. South Carolina had commenced to mutter the maxims of her political heresy, which precipitated soon after the rupture between the President and the Vice-President, Mr. John C. Calhoun.

With even a cursory glance at the condition of the medical profession in the United States, we discover that great advance had been made since the first resident physician in the country, Dr. Walter Russell, came from England to the Colony of Virginia in 1608. Drs. John Bard and Peter Middleton had, in 1750, been first to inject and dissect the body of a criminal for anatomical purposes; and in fifteen years thereafter the Medical Department of the University of Pennsylvania had been organized—the pioneer of all the medical colleges in the country. The profession venerated the name of the heroic Dr. Warren, who fell at the battle of Bunker Hill, as well as that of Dr. Benjamin Rush, who was one of the signers of the Declaration of Independence. Dr. Physick had invented the tonsillotome which is now in general use, and established his reputation as one of the most eminent surgeons in the United States. Dr. McDowell, in 1809, had performed ovariotomy, and lithotomized the poor lad who subsequently became President, James K. Polk. Operations had been recorded for ligation of the carotid, subclavian, brachial, femoral, internal, external and common, iliac arteries; amputations had been accomplished at the hip and shoulder joints; the radius, clavicle, head of the humerus and femur, the astragalus, and the fifth and sixth ribs had been exsected; the tumor of spina bifida, the tongue, the spleen and the parotid gland had been excised; lithotripsy and staphyloraphy had been done; the hydrocephalic head had been tapped.

Thirty-two medical works* had been issued from the American press—some of them, translations from foreign authors; some, reprints of foreign editions; some, from the pen of native-born physicians and surgeons. Thirty medical periodicals had been established, but, at the date to which I refer, of these, but ten had survived.†

* See the Principles and Practice of Surgery, by Henry H. Smith, M.D., Phil. 1863, from which these details have been obtained. The works of American authorship referred to, are: Review of Medical Improvements in the 18th Century, by David Ramsey (1800); Martin on Goitre (1800); Barnwell's Causes of Disease in Warm Atmospheres (1802); Parrish on Ruptures (1811); Dorsey's Elements of Surgery (1813); Hosack's Surgery of the Ancients (1813); Mann's Medical History of the Campaigns of 1812–14 (1816); Anderson's System of Surgical Anatomy (1822); Gibson's Institutes and Practice of Surgery (18[illegible]4); Barton's Treatment of Anchylosis by Formation of Artificial Joints (1827); Darrach's Anatomy of the Groin (1830); and Gross's Anatomy, Physiology and Diseases of Bones and Joints (1830).

† These survivors were: Transactions of the College of Physicians of Phila., 8vo, Phil. (1793—1850); North American Medical and Surgical Journal, Phila. (1826—1831); American Journal of the Medical Sciences, 8vo,

### CHICAGO IN 1831.

3 FORT DEARBORN. 2 Dr. WOLCOTT. 1 JOHN KINZIE.

The county of Cook, in Illinois, was organized in the year 1831, and that may properly be considered the date of the commencement of the medical and general history of Chicago.* For a description of the place at that time, I am largely indebted to the work of Mrs. John H. Kinzie, to which reference has been made.

The fort was enclosed by high pickets, with bastions at the alternate angles, and large gates opening to the north and south; while here and there were small sally-ports for the accommodation of the inmates. Beyond the parade ground, which extended south of the pickets, were the company gardens, well filled with currant bushes and young fruit trees. The fort itself was stationed on the south bank of the river, near what is now its mouth, but, at this time, the river itself swept around the little promontory on which the stockade was erected, and, passing southward nearly beneath what is now the pavement of Michigan avenue, joined the lake at a point about half a mile below, where Madison street now extends. The left bank of the river was formed by a long sand-spit, extending southward from the northern shore. This was cut through by the engineers of the United States in 1833, for the purpose of improving the harbor; and thus was formed the present river-mouth. The old fort stood like a faithful sentinel at his post till 1856, when it was demolished, after having witnessed the growth of its *protégé* into the encroaching city that enforced its destruction.

Between the gardens and the river bank was a log cabin, erected in 1817. It had been the residence of Jean

Phila. (1827—1876); Boston Medical and Surgical Journal, 8vo, (1828); Transylvania Journal of Medical and Associated Science, Lexington, Ky. (1828–37); New York Medical and Physical Journal (1829–31); Maryland Medical Recorder, 8vo, Baltimore, Md. (1829–32); New York Medical Inquirer and American Lancet (1830); and the New York Medico-Chirurgical Bulletin (1831–2).

* The map of the original town, by James Thompson, surveyor for the State Canal Commissioners, is dated Aug. 4, 1830. It provided for a public levee from South Water street to the river, the plan of which was subsequently abandoned.

Baptiste Beaubien, Point au Sable, a native of San Domingo, who located here in 1796, and thus occasioned the utterance of the Indian Hibernicism that "the first √ white man in Chicago was a negro." The cabin had finally come into the possession of an Indian trader, named Le Mai, from whom it had been purchased by Mr. Kinzie. Further to the south was a rickety tenement, built several years before by John Dean, a post-sutler, and now used by his family as a school-house and residence. It had been so far undermined by the lake as to have partially fallen backward.

On the northern bank of the river, and directly in front of the fort, stood the residence of Mr. John Kinzie. It was a long, low building, with a piazza extending along its front, overlooking a broad, green space which stretched between it and the river. It was shaded by a row of √Lombardy poplars in front, and two immense cottonwood trees in the rear; a fine and well-cultivated garden showing on one side, with dairy, stables and other outhouses adjacent.

Still further to the north, stood a small but substantial building of hewed and squared logs, known as the Agency House. On either side of its two wings were the residences of the Government employees—blacksmiths and laborers—mostly half-breed Canadians, with an occasional Yankee among them. There was but one other building on the North Side, and that was at this time vacant. It had been erected by a former resident, named Samuel Miller.

On the southern bank of the river, between the fort and the point where the river divides, there was no dwelling house. The prairie here was low and wet—in the driest weather affording a poor foot-path for the pedestrian, and often overflowed in the rise of the river water. Mrs. Kinzie states that a horseman who once made the trip had gotten his feet wet in the stirrups, and declared that he "would not give a sixpence for an acre of it." A muddy streamlet wound around from the present site of the Tremont House, to join the river at the foot of State street.

The projection of land between the north and south branches was variously known as "The Point," "The Forks," or "Wolf Point"—the latter term having been derived from the name of an old Indian chief. Here was a canoe ferry for the accommodation of passengers. The residence of Mark Beaubien, Jr., distinguished by its additional upper story and bright blue window shutters, stood upon the Point, and was the admiration of the little community in consequence of these modern improvements. Facing down the river from the west, was a small tavern, kept by Mr. Elijah Wentworth, and near it lay several log cabins, occupied by Alexander Robinson, the half-breed Pottawatomie chief, his wife's connections, Billy Caldwell, the "Sau-ga-nash," and the wife of the latter, who was the daughter of "Nee-scot-nee-meg." Gholson Kercheval, a small trader, occupied one of these cabins, and, in close proximity, stood the school-house, a small log cabin, used occasionally as a place of public worship. Here, we learn that a reverend gentleman named Charles See did violence to the King's English on Sundays when opportunity offered. Some distance up the North Branch, was located the Clybourn residence, and an old building, erected some time before by a settler named Reuben E. Heacock, was still standing, at a point four miles distant up the South Branch. This house had some interest attaching to it, in consequence of its connection with the old Indian massacre.

At the time to which we refer, the fort was occupied by two companies of soldiers, under the command of Lieutenant Hunter, in the absence of Major Fowle and Captain Scott. Lieutenant Furman had died during the preceding year. The subordinate officers were Lieutenants Engle and Foster. The Kinzie family then occupied the Agency House, and Post-Master Bailey was quartered in their residence.

In the brief description above given are enumerated, it is believed, all the buildings then erected, and all the residents occupying them, with the single exception of Dr. Harmon, to whom we hasten to give our attention.

Elijah Dewey Harmon was born on the 20th day of August, 1782, in the town of Bennington, Vermont. After completing his education as far as possible in that place, he resorted to Manchester, in his native State, where he pursued the study of medicine in the office and under the direction of a noted practitioner of the place, named Swift.* At the expiration of the two or three years which were employed in acquiring a knowledge of his profession, he removed to Burlington, Vt., at the early age of twenty-five years, and began to practice medicine in connection with the business of a drug store, as was customary at that time.† Here he remained until the occurrence of the war of 1812, when he hastened to offer his services as a volunteer surgeon. Dr. Harmon, during this period, had the distinguished honor of serving as a surgeon on board the flag-ship of the gallant Commodore McDonough, in the battle of Plattsburgh, on the 11th day of September, 1814. If the terrific fire to which the *Saratoga* was exposed in that engagement be remembered, we may well believe that the doctor's skill and courage must have been put to a severe test.

At the close of the war, the doctor returned to Burlington, where he continued in civil practice with a success which contributed not only to his financial prosperity, but to the establishment of his reputation. In the year 1829, however, he suffered some pecuniary losses in consequence of his speculations connected with a marble quarry, and he determined, as many of his successors

* The three medical schools of Vermont had not then been founded. Castleton Medical College was established in 1818 ; the Medical Department of the University of Vermont in 1822; and the Vermont Medical College in 1827.

† I am indebted for these details to his son, still a resident of Chicago, Mr. I. D. Harmon. Unfortunately, most of the family documents were destroyed in the Great Chicago Fire, and among them was the diploma of the University, which conferred upon the doctor his degree in medicine.

have done since then, to advance his fortunes in the far West. During that year, therefore, he spent several months in Jacksonville, Ill., engaged in the selection of a suitable locality in which to settle. After returning to his native State and completing his arrangements for a final removal, he left a second time, and proceeded directly to Chicago, traveling on horseback from Detroit, and arriving here in the fall of 1830. His family joined him in June of the succeeding year.

It happened that Dr. J. B. Finley, the surgeon of the garrison, was, at this time, about to leave the post, and thus Dr. Harmon came to be at once installed in his position—he and his family taking up their residence in the fort, which then was held by two companies of United States troops. Little must have occurred to disturb the monotony of his new duties, until the succeeding spring, when the country became agitated again in consequence of the Black Hawk war.

In May of the year 1832, cholera made its appearance upon the New England coast, and extended rapidly westward along the water courses of our northern frontier, one branch apparently diverging by way of the Hudson river to New York City. Five companies were at once hurried, in consequence of the exigencies of the time, from Fortress Monroe to Chicago, and traversed the entire distance of 1,800 miles in eighteen days, a transportation which was then considered unprecedented in rapidity, and which was really marvellous in view of the facilities then attainable. General Scott arrived with this detachment in a steamer,* on the eighth day of July, 1832, and, as might have been expected, cholera rapidly spread through his command, one man out of three being attacked, and many dying.

It was then wisely decided to separate the two companies in the fort from those which had newly arrived, and thus, if possible, prevent the extension of the dis-

* This vessel, the Sheldon Thompson, was the first steamer to visit Chicago, but it did not enter the harbor.

ease among the former. These two companies, accordingly, were encamped at a short distance from the stockade, and placed under the professional charge of Dr. Harmon. While due allowance is, of course, to be made for the favorable circumstances in which this isolated detachment was placed, it certainly reflects great credit upon their surgeon, that among the men affected with cholera under his charge, but two or three deaths occurred. It may be here remarked that the doctor attributed his success to the fact that he did not employ calomel in the treatment of the disease. Of the treatment employed in the fort, and its results, we shall have something to say hereafter.

Some misunderstanding seems to have occurred at this time between General Scott and Dr. Harmon, in reference to the line of conduct pursued by the latter. The general, like a great many military men since his day, desired the surgeon to devote his attention exclusively to the companies under his care, while the good-hearted doctor could not but heed the demand for his services by civilians, and others not in the military service. Certain it is that he endeared himself to the citizens of the little town by his conduct at this time, and we are not surprised to learn that after the epidemic had subsided, Gen. Scott and his command had pushed farther south, and the monotonous routine of garrison life had been endured for another year, that in the spring of 1832, Dr. Harmon, having secured the Kinzie house as a place of residence, removed to it with his family.

Before concluding, however, the narrative of Dr. Harmon's military career, it is proper to mention the fact that he performed an amputation in the fort during the winter of 1832. This is certainly the first record that we possess of any capital operation in Chicago; and it is probable that it was, in point of fact, the first surgical operation of any magnitude ever attempted in the place. A half-breed Canadian had frozen his feet, while engaged in the transportation of the mail on horseback from

Green Bay to Chicago.* The doctor, assisted by his brother, tied the unfortunate man to a chair, applied a tourniquet to each lower extremity, and with the aid of the rusty instruments which he had transported on horseback through sun and shower from Detroit to Chicago, removed one entire foot and a large portion of the other. Needless to say those were not the days of anæsthetics, and the invectives in mingled French and English, of the mail carrier's vocabulary, soon became audible to every one in the vicinity of the stockade. It is gratifying to note that the first recorded amputation in Chicago was crowned with a most satisfactory success.

Dr. Harmon may properly be called the Father of Medicine in Chicago. For, in the removal and establishment of himself and his family in the Kinzie house, we find the first trace of the settlement of a civil practitioner in the community. His object in effecting this change was to engage in the practice of medicine—all other transactions having been made subordinate to this.

A brief glance at his surroundings at this time might prove interesting. His office and residence combined was a cabin, whose floor and walls were constructed of hewn logs—the former, of course, innocent of carpets. It contained twelve rooms, lighted by small panes of glass, and heated by wood burned in stoves brought from Detroit. His food was largely bacon, transported from the valley of the Wabash in the now traditional "prairie schooner," with lard as a substitute for butter—and an occasional slice of venison, or a wild turkey, as an *entremets*. His medicines he had brought with him from Vermont, together with the rusty instruments of which mention has been made. But his medical library —to his honor be it said—was the chief part of his armamentarium. It consisted of over one hundred volumes, and some of those have, without doubt, been enumerated in the foot note upon another page giving the list of

* The winter of this year was unprecedentedly severe. There is abundant collateral evidence on this point.

works published in America before this date. How many of his successors have engaged in the practice of medicine, with far less provision for the refurnishing of the storehouse of professional science!

The doctor's visits must have been made largely on foot; as Beaubien is reported to have possessed the only vehicle on wheels to be found in the town,* and that judging from the description, must have greatly resembled the "one-hoss shay," so graphically delineated by another member of our profession. When he had occasion to cross the river, it was necessary to paddle himself over, in one of the dug-out canoes, which were generally tied in front of each residence, or resort to "Wolf Point," where a canoe ferry offered merely the same facilities.

Some idea may be formed of the general character of the doctor's patients, from a criticism written by Latrobe in the autumn of 1833.† He describes "a doctor or two, two or three lawyers, a land agent and five or six hotel keepers; these may be considered the stationary occupants and proprietors of the score of clap-board houses around you; then, for the birds of passage, exclusive of the Pottawatomies, you have emigrants, speculators, horse dealers and stealers; rogues of every description, white, black and red; quarter-breeds and men of no breed at all; dealers in pigs, poultry and potatoes; creditors of Indians; sharpers; peddlers; grog-sellers; Indian agents, traders and contractors to supply the Post"—certainly not a highly encouraging picture of a *clientèle*.

Medical examinations for life-insurance, which have since proved a source of remuneration to the profession, were then unknown. It would appear from an article

* It is said that the villagers, upon the arrival of this vehicle from the East, paid it distinguished honor, "turning out in procession and parading the streets."—*Chicago Antiquities*. No. 2.

† Western Portraiture and Emigrants' Guide. Daniel S. Curtis. New York. 1852.

published during the ensuing year in a literary periodical, not only that the general subject of life insurance was little understood in the West, but that the basis upon which policies were issued to the assured, was the statement of the applicant, endorsed by his family physician only.*

As for the fees given in remuneration of professional services, perhaps the less said upon the subject the better. But it is pleasant to note that a precedent had been established in the country, for the encouragement of the humble toilers on the Lake shore. Dr. McDowell had even then received fifteen hundred dollars for the performance of ovariotomy†—a reward which, considering the scarcity of money and the price of labor and food, was fully equal to the famous fee paid Sir Astley Cooper by Mr. Hyatt, and only surpassed by the munificent honorarium, given to a contemporary surgeon as recently reported in the secular press.

Mrs. Kinzie describes the doctor as she used to see him, when she and her friends made little excursions on horseback in the vicinity of their residence.‡ On one occasion he was engaged in superintending the construction of a sod fence near the lake, and planting fruit stones, with a view to a prospective garden and orchard, under the branches of the trees that arched overhead. "We usually stopped," she remarks, "for a little chat. The two favorite themes of the doctor were, horticulture and the certain future importance of Chicago. That it was destined to be a great city, was his unalterable conviction, and indeed, by this time, all forest and prairie as it was, we half began to believe it ourselves."

"The glorious dreams of good Dr. Harmon," as they were called, produced a practical result in his case. In the spring of 1833, he secured by pre-emption, one hun-

* See the Western Monthly Magazine, Vol. 2, 1834. Cincinnati, Ohio.

† Lives of Eminent American Physicians and Surgeons ,of the 19th Century. S. D. Gross, M.D. Philadelphia. 1861. Page 228.

‡ Opus cit.

dred and thirty acres of land lying next to the Lake and just south of what is now 16th street. In order to make good the title, he built a small log cabin upon this property, and resided there until the spring of 1834, when he left the State for Texas. To-day the doctor's farm is worth between five and six millions of dollars.* Had his sons possessed the same confidence in the future of Chicago as that felt by their father, they would now be enjoying the fruit of his wise providence. One of them, however, had been entrusted with a power of attorney for the sale of this property, and accordingly, contrary to the advice and counsel of its pre-emptor, it was sold for a sum which then seemed an enormous price for the land, but which was in fact a paltry consideration for the magnificent squares which are now covered by elegant metropolitan residences. It is, however, somewhat gratifying to reflect that the most valuable residence property in Chicago, was once, in fee simple, the homestead of its earliest resident physician.

Dr. Harmon died on the 3rd day of January, 1869, after having made several trips to Texas, where he not only engaged in the practice of medicine, but invested in real estate which has since greatly appreciated in value.

It will be seen from what has preceded, that he was of an adventurous disposition—an essential element in the character of all successful pioneers. A recent historiographer has said that the early settlers of the West made the name adventurer forever respectable—and he has wisely spoken. Out of their loins came a commonwealth—most of its virtues are hereditary, and its vices have been chiefly acquired.

Dr. Harmon, during his life, served not only as a Justice of the Peace, but, in conjunction with Col. R. J. Hamilton and Mr. Russell E. Heacock, officiated in the first Board of School Commissioners, organized under the law. The Doctor's strong conviction of the immense prospective value of the land known as the School Sec-

* This is the value as estimated by W. D. Kerfoot, Esq., of Chicago.

tion, led him here also to strenuously oppose its sale. In this matter, as in the disposition of his own property, his judgment was overruled by others, and but forty thousand dollars were for this reason realized from the sale of six hundred and forty acres of land, the value of which to-day is more than fifty millions of dollars.

In person, Dr. Harmon possessed a commanding figure, and his features were such as proclaimed at a glance both his parentage and his profession. There were the strong outlines of the New England face, with the beard shaven in the manner adopted by the profession in France—a face whose like is often seen in the portraits of the heroes of the Revolution. There were, besides, the evidences of broad culture, high attainments and wide experience—the traits of one, whose mental horizon is not bounded by the definitions of other men. He was also a gentleman having a generous, whole-hearted disposition. One of the streets of our city still bears his name. The profession have little need to be ashamed of their first civil representative in Chicago.

In order to a correct understanding of this narrative, it is now necessary to retrace our steps to the old fort, which we left at the time of the exodus of Dr. Harmon and his family. In response to my inquiries (for the answers to which I am greatly indebted to Assistant Surgeon John S. Billings, U. S. A., now of the Surgeon General's Office,) it is made clear that there is no record of any medical officer stationed at the fort, prior to the time of Assistant Surgeon S. G. J. DeCamp, of New Jersey. Of Dr. Van Voorhees and Dr. J. B. Finley, no information can be obtained at the War Department. Dr. DeCamp was appointed Assistant Surgeon, October 10, 1823 ; promoted Surgeon, December 1, 1833 ; retired in 1862, and died at Saratoga Springs, New York, September 8, 1871. As it is he who makes the official report of the cholera cases in the fort, during the prevalence of the epidemic,*

* Statistical Report on the Sickness and Mortality in the Army of the United States, prepared under the direction of Thomas Lawson, M.D., it seems probable that it was he who was present and responsible for the treatment and its results. According to this report, two hundred cases were admitted into hospital in the course of six or seven days, out of the entire force of one thousand, fifty-eight of which terminated fatally. All the cases were treated by calomel and blood-letting, and, according to Surgeon DeCamp, this proved so efficacious in his hands, that he regarded the disease as "robbed of its terrors" (!). He inclines to the opinion that the disease was contagious, in consequence of the fact that several citizens of "the village" died of cholera, although, prior to the arrival of the steamer, no case had occurred, either in the fort or the village. He notes the predisposition to the disease, evident in those of intemperate habits.

The table which is appended in a note,* is compiled from reports of each quarter of the year, published in the volume referred to above. Although it is a return from a military garrison, it is interesting as it is probably the first contribution to vital statistics ever prepared in Chicago.

The inhabitants of the little town did not soon forget

Washington, 1840. This appears to be the first of the brilliant series of publications issued from the Surgeon General's office ; and I am indebted for this, also, to the kindness of Assistant Surgeon John S. Billings, U. S. Army.

* Abstract exhibiting principal diseases at Fort Dearborn for ten years:

| Years | 1829. | 1830. | 1831. | 1833. | 1834. | 1835. | 1836. | TOTALS. |
|---|---|---|---|---|---|---|---|---|
| Mean Strength | 91 | 90 | 92 | 104 | 91 | 96 | 104 | 668 |
| DISEASES: | | | | | | | | |
| Intermittent Fever | 17 | 18 | ----- | 19 | 32 | 19 | 31 | 136 |
| Remittent Fever | ----- | 15 | 1 | 1 | 2 | 5 | 2 | 26 |
| Synochal Fever | ----- | 1 | 1 | ----- | ----- | ----- | ---- | 2 |
| Diseases of Respiratory Organs | 11 | 8 | 1 | 10 | 22 | 14 | 23 | 89 |
| Diseases of Digestive Organs | 30 | 22 | 9 | 69 | 84 | 53 | 42 | 309 |
| Diseases of Brain and Nervous System | 2 | 3 | ----- | ----- | 3 | ----- | 1 | 9 |
| Rheumatic Affections | | 10 | 3 | 7 | 3 | 7 | 15 | 51 |
| Venereal Affections | | 1 | 3 | ----- | ---- | ----- | 2 | 7 |
| Ulcers and Abscesses | 16 | 12 | ----- | 9 | 8 | 5 | 7 | 57 |
| Wounds and Injuries | 19 | 15 | 10 | 41 | 19 | 10 | 14 | 128 |
| Ebriety | 4 | ----- | ----- | 11 | 2 | 4 | 8 | 29 |
| All other Diseases | 12 | 5 | 2 | 26 | 10 | 20 | 15 | 90 |
| TOTALS | 118 | 119 | 30 | 193 | 185 | 137 | 160 | 933 |

The post was unoccupied during the year 1832, and abandoned in 1840

the ravages of the epidemic which had visited them. After a year had elapsed, the boatman who paddled up the river in his dug-out canoe, could perceive the ends of the bark coffins* projecting from the sand hills on the right bank, and even occasionally note their exposed contents.

The next medical incumbent at the fort was Dr. Philip Maxwell,† who was born at Guilford, Windham county, Vt., on the 3d of April, 1799. He studied medicine with Dr. Knott of New York City, but took his degree in one of the Medical Universities of his native State.‡ He commenced the practice of his profession in Sackett's Harbor, New York, but temporarily abandoned it when elected a member of the State Legislature. In the year 1832, he was appointed an Assistant Surgeon in the U. S. Army, and was first placed on duty in Green Bay, Wisconsin. He was ordered to report at Fort Dearborn on the 3rd day of February, 1833, and arrived here on the 15th of the next month, remaining until official orders were received for the discontinuance of the post, on the 28th of December, 1836. During the time in which he was on duty in camp at Wisconsin, he was so impressed with the beauty of the country in the neighborhood of Geneva Lake, that he subsequently purchased the entire township, and it is now the seat of the elegant homestead of his family descendants. He was promoted to the Surgeoncy, July 7, 1838, and subsequently served with Gen. Zachary Taylor, at Baton Rouge, and on the St. John's river in Florida. Like Dr. Harmon, he became a civil practitioner in Chicago after resigning his commission, and from 1845 to 1855, was in partnership

* These are erroneously reported as "uncoffined," in The History of Illinois from 1673 to 1873, by Alexander Davisson and Bernard Stuvé, Springfield, Ill., 1874. It is probably true, however, that the sepulture was often as hasty and informal as there described

† The information given above has been obtained through the kindness of his son-in-law, Mr. J. C Walter, of Chicago.

‡ The names of these institutions, with the date of their foundation, wil be found in a note upon page 206.

with Dr. Brockholst McVickar, who is still engaged in the practice of medicine in this city.

Dr. Maxwell had such a physique as one can admire to-day in some of the older of our army officers. He was straight and portly in figure, six feet and two inches in height, two hundred and seventy-five pounds in weight. For all this, according to Mr. B. F. Taylor, who has drawn several pictures of early Chicago in his graphic and entertaining style, "his step was as light as that of a wisp of a girl." Judge Caton still remembers his appearance in the year 1836, when engaged in dancing at a ball dressed in full regimentals with epaulets. On this occasion his partner was one of the servant-maids of his host. Whether this occurred through inadvertence or in consequence of the well-known scarcity of ladies in the early days on the frontier, may not perhaps be determined. Hoffman is also supposed to refer to Dr. Maxwell in his characteristic account of one of the first balls given in Chicago, when he describes "the golden aiguilette of a handsome surgeon, flapping in unison with the glass beads upon a scrawny neck of fifty."*

Dr. Maxwell died on the 5th of November, 1859, aged 60 years. His name will ever be honored in Chicago as the second in its line of medical succession; and his portrait may still be seen with those of the twelve gentlemen who are counted among its oldest residents."†

Long before Dr. Maxwell settled in private practice, the development of the town had induced other physicians to engage in professional business within its limits. This development, however, was at first feeble and protracted. At the time of the sale of land by the commissioners in 1830, the town lots, eighty by one hundred

* Winter in the West. Charles Fenno Hoffman. 1834.

† This picture was taken by the photographer, A. Hesler, in 1856. It includes the faces of Wm. B. Ogden, the first mayor of Chicago, J. H. Kinzie, Mark Beaubien, Geo. W. Dole, Jacob Russell, B. W. Raymond, G. S. Hubbard, Jno. P. Chapin, Philip Maxwell, Wm. B. Egan, and others.

and sixty feet, sold for between forty and sixty dollars. In the year 1832, the assessment for taxes amounted to but $357.78; and the first public improvement was an estray pen, erected on the site of the present Court House at an expense of twelve dollars. Not many vessels had entered the harbor, since the schooner Marengo, foremost of a mighty fleet, floated into the river from Detroit in 1831.* It was not indeed till the year 1834, that one could see any arrangement of houses in such an order as to form a street. And yet, at that date, there was a marked increase in the population, according to the figures given in a Gazetteer of the State, then published.† It was estimated that there were one thousand inhabitants of the town—an increase of nearly eight hundred since the preceding year. There were "three houses for public worship, an academy, *an infant* and other schools, twenty-five or thirty stores, some of them doing a *large* business, several taverns, and a printing office."‡

Of the physicians who succeeded those heretofore noticed, space forbids much more than a passing mention. In an address delivered before the Rock River Medical Society, at the time of its organization,§ Dr. Josiah C. Goodhue spoke as follows: "Dr. Harmon was the pioneer among the medical faculty of this corner of Illinois; Dr. Kimberly was the second; then came Dr. Jno. T. Temple; Dr. Clark next; Drs. Egan, Eldridge and myself soon followed, at about the same time. This brings us to the spring of 1834, when a perfect flood of emigration poured in, and with it a sprinkling of doctors. Prior to 1840, nine-tenths of all the physicians who had located themselves in this region, had done so with reference to pursuing agriculture, and with the avowed intention of abandoning medical practice; most

* See Reynolds' Sketches, op. cit.

† A Gazetteer of Illinois; J. M. Peck, Jacksonville, 1834.

‡ The Chicago Democrat—established by John Calhoun.

§ Illinois and Indiana Medical and Surgical Journal, Vol. 2, p. 260.

of whom, either from the necessities of the case, or from finding more truth than poetry in pounding out rails, resumed their profession and divided their attention between farming and medicine." In the last sentence, Dr. Goodhue of course refers chiefly to practitioners settling in that part of the country where the Rock River Medical Society proposed to hold jurisdiction.

Of the physicians named above, only one is now living, Dr. Eldridge, who resides at Naperville, Ill.; but all were more or less known to many of the citizens of Chicago who have survived them. Dr. Jno. T. Temple, who removed to the city in 1833, was a graduate of Middlebury College, Castleton, Vt., (Dec. 29, 1830), and seems for a time to have done duty as a volunteer surgeon of the garrison. So far as is known, he should be credited with the performance of the first autopsy made in the city, as well as with the rendition of the first medico-legal testimony in court. An Irishman had been indicted for murder; and Dr. Temple was summoned to make a post-mortem examination of the victim. The ease with which he separated by a few skillful touches of his knife, the bones concerned in the sterno-clavicular articulation, is still remembered by those who witnessed the unusual spectacle. The attorney for the defense, however, on this occasion, succeeded in proving that his client had been guilty of manslaughter, and in securing his acquittal on the ground that he was innocent of murder as charged in the indictment! In comparing the two professions, as they here appear in their representatives, it may be fairly inferred that the anatomical knowledge of the expert was more than equal to the legal acumen of the judge!

Dr. Temple, soon after, secured a contract from the Postmaster General, Amos Kendall, for carrying the mail between Chicago and Ottawa. He obtained an elegant, thorough-brace post carriage from Detroit, which was shipped to this port via the lakes, and, on the 1st of January, 1834, drove the first mail coach with his own hand from this city to the end of the route for which he had received a contract. On this first trip, he was accompanied by the Hon. Jno. D. Caton, to whom I am greatly indebted for many of these details. The demand for this accommodation could not then have been very great, as there was *no mail matter for transportation in the bag carried on this first trip!* *

Dr. William Bradshaw Egan was born "on the banks of the beautiful Lake of Killarney," September 28, 1808, and was the second cousin of Daniel O'Connell, whose name has already appeared in these pages. His medical studies were begun with Dr. McGuire, a surgeon in the Lancashire collieries, but were also pursued in London and in the Dublin Lying-in-Hospital.† After his arrival in this country, he was licensed as a physician by the Medical Board of the State of New Jersey, in the spring of 1830, and began his professional career in Newark and New York, having been associated in the latter city, with Prof. McNeven and Dr. Busche. Here also he was married to Miss Emeline M. Babbatt, who accompanied him to Chicago in the fall of 1833. In the year 1846, he purchased for three dollars per acre, the beautiful property in the West Division of the city, comprising three and one-half acres, which is to-day the residence of his family; and also laid out his farm—Egandale Park, on the Lake Shore, about six miles distant from the Court House. At one time he was also in possession of the land upon which the Tremont House now stands. During the sessions of 1853-4, he was a member of the lower house of the State Legislature; and also during his lifetime served as recorder of the city and county.

Dr. Egan was, as has been often remarked, a perfect specimen of the "fine old Irish gentleman." He had a noble presence and a commanding figure; but that which especially attracted his associates, was his exuberant

* Dr. Temple is said to be now living in St. Louis, and engaged in homœopathic practice.

† Chicago Magazine, Vol. 1, No. 3; May, 1857.

fancy, his sparkling wit and his keen perception and graphic delineation of the ludicrous.

He not only established an excellent professional reputation in Chicago, but was much esteemed socially; not more so, however, than his wife, whose graces of person and character were the admiration of the circle in which they both moved. Mr. Joseph Grant Wilson, in some sketches recently published in Appleton's Journal, describes the doctor, as he once appeared after the girth of his saddle had given way during a wolf hunt, and his full-blooded Kentucky racer had left him: "standing on the prairie, a large fur cap on his head, an enormous Scotch plaid cloak (purchased at the 'store' of Mr. G. S. Hubbard) belted around his Brobdignagian waist, and shod with buffalo overshoes." It is of Dr. Egan that the story is told which has lately been revived and gone the rounds of the medical press. He had engaged extensively in the purchase and sale of real estate, the conditions of transfer at that day being generally dependent on what was known as "canal time." It is said that the doctor having been, on one occasion, asked by a lady who was his patient, how she should take the medicine ordered for her, the response was: "a quarter down and the balance in one, two and three years"! At the time of the first breaking of ground for the construction of the Illinois and Michigan Canal, on the 4th of July, 1836, Dr. Egan was selected to deliver the oration; and this is only one of several evidences of his great popularity. We find the beauty of his garden and his genial hospitality extolled in complimentary terms in a work which appeared a few days before the date of his death.* This event occurred in 1856.

Dr. Josiah C. Goodhue came to Chicago directly from Canada, but was the son of an American physician, the first president of the Berkshire County Medical College,

* Summer Rambles in the West. Mrs. Ellet. New York. 1853.

of Pittsfield, Mass.* He enjoyed a very large and lucrative practice while residing in this city, but subsequently removed to Rockford, Ill., where he died later in consequence of an accident. Drs. Stuart and Lord were among the physicians first succeeding those enumerated above—the former having enjoyed the reputation of being the Beau Brummel of the profession, and the latter having distinguished himself by securing a patent for a labor-saving pill machine.

Dr. John H. Foster came to Chicago in 1835, and died here on the 18th day of May, 1874.

It would be unjust in this connection to leave unmentioned the name of the first druggist in Chicago. Mr. Philo Carpenter was a native of Massachusetts, born on the 27th day of February, 1805. In the year 1827, he commenced the study of medicine which he prosecuted for two or three years under the direction of Dr. Amatus Robbins, of Troy, New York. He arrived in Chicago in the month of July, 1832, just at the time when the cholera-stricken troops under the command of Gen Scott, had been transported to the fort. Mr. Carpenter had abandoned his medical studies in order to pursue the more congenial business of an apothecary, but in the present emergency, he attended many cases of cholera, and rendered an assistance which was very highly appreciated. Soon after, he opened a drug and general store in a small log cabin near the eastern end of the present Lake Street bridge, from which, as his business increased, he removed into a more pretentious frame building. In the spring of 1833, Dr. Edmund Stoughton Kimberly, of Troy, N. Y., alluded to in Dr. Goodhue's address, in company with Mr. Peter Pruyne, opened a second druggists' establishment. Dr. Kimberly was registered in the year 1833, among those who voted for the incorporation of the town. He died at his late

* Extracts from Journal of Rev. Jeremiah Porter ; recently published in the Chicago Times.

residence in Lake County, Illinois, Oct. 25, 1874, aged 72 years.

Without pausing to comment further upon the history of the medical gentlemen who rapidly succeeded those already mentioned, I hasten to present a brief sketch of the remarkable man, who, perhaps to a greater extent than any of his professional peers in Chicago, achieved a national reputation. Through the kindness of the Hon. Edward Huntington, of Rome, N. Y., I have obtained access to some notes prepared on the subject by Calvert Comstock, Esq., from which the subjoined details have been in part supplied.

Daniel Brainard was born on the fifteenth day of May, 1812, in the town of Western,* Oneida Co., N. Y. His father, Jepthai Brainard,† was a farmer in comfortable pecuniary circumstances and of excellent character, while his mother was a most exemplary woman, whose influence was deeply impressed upon her children, and doubtless did much in awakening the genius and inspiring the aims of the son in his early life. He was given a good common school and academic education, which laid the foundation for that exact and exhaustive method of investigation which characterized his subsequent professional studies. Having chosen the profession of medicine, he entered the office of Dr. Harold H. Pope, a distinguished physician and surgeon of Rome, N. Y., pursuing his studies also in Whitesboro, and New York City, and obtaining his degree of Jefferson College, Philadelphia, Pa., in the year 1834.

* In some biographical notices, the place of his birth is erroneously stated to be Whitesboro, in the same county.

† In a Genealogy of the Brainard Family by the late Rev. David D. Field, 1857, it appears that the first individual who bore the [illegible]me in America, was a Daniel Brainard, of Haddam, Ct. (1662). But, according to Mr. Hurlbut, in whose possession the volume is, in spite of the industrious labors of Mr. Field, the materials it contains are so wretchedly arranged, misplaced and mystified, that the work is of comparatively little value; and it is almost impossible to trace with any clearness the line of ancestry, from the records there given.

During this preparatory career he delivered some lectures of a scientific character in Fairfield, N. Y., and in the course of the two years succeeding his admission to the profession, he delivered another series of lectures on anatomy and physiology in the Oneida Institute. He commenced the practice of medicine in Whitesboro, N. Y., whither his family had removed from the farm in Western, on account of the educational advantages afforded in the former place. Here he remained for some two years in partnership with Dr. R. S. Sykes,* a gentleman who had directed his medical studies before his departure from the village.

Henry H. Hurlbut, Esq., of Chicago, who has kindly furnished several facts of interest in this connection, informs me that he was recently shown by a lady a small quarto volume which affords a glimpse of the literary annals of the little village. It is the record of proceedings of the "Mæonian Circle"—composed of young ladies and gentlemen—and contains the signature of Dr. Brainard as an officer of the Club in the autumn of 1834. Among the names of members appears that also of Miss F. M. Berry, the authoress of the "Widow Bedott Papers."

Soon after this, Dr. Brainard determined to remove to the West. His advent and earliest history in Chicago, are best described in the language of the Hon. J. D. Caton, to whom I have already had occasion to express my obligations for valuable aid in the preparation of this sketch:

"About the first of September, 1835, Dr. Brainard rode up to my office, wearing pretty seedy clothes and mounted on a little Indian pony. He reported that he was nearly out of funds, and asked my advice as to the propriety of commencing practice here. We had been professional students together in Rome, N. Y., when he was in the office of Dr. Pope there. I knew him to have been an ambitious and studious young man,

* Dr. Sykes is said to be now living in Chicago, aged 80 years.

of great firmness and ability, and did not doubt that the three years since I had seen him, had been profitably spent in acquiring a knowledge of his profession. I advised him to go to the Indian camp where the Pottawattomies were gathered, preparatory to starting for their new location west of the Mississippi river, sell his pony, take a desk or rather a little table in my office, and put his shingle by the side of the door, promising to aid him as best I could in building up a business. During the first year, the doctor's practice did not enter those circles of which he was most ambitious. Indeed it was mostly confined to the poorest of the population, and he anxiously looked for a door which should give him admission to a better class of patients. While he answered every call, whether there was a prospect of remuneration or not,* he felt that he was qualified to attend those who were able to pay him liberally for his services. At length the door was opened. A schooner was wrecked south of the town, on which were a man and his wife, who escaped with barely their clothes on their backs. They were rather simple people, and belonged to the lowest walks of life. They started for the country on foot, begging their way, and, when distant some twelve miles, encountered a party of men with a drove of horses, one of whom pretended he was a sheriff, and arrested them for improper purposes. When they were set at liberty, they returned to the town, and came to me for legal advice, the woman being about five months advanced in pregnancy. I commenced a suit for the redress of their grievances, and the doctor took an active interest in their welfare. He procured for them a small house on the North Side, and made personal appeals to all the ladies in the neighborhood, for provision for their needs. Mrs. John H. Kinzie become particularly interested in their case, and paid frequent visits to the cabin with other ladies. The nervous system of the

* Dr. J. W. Freer informs me that this was true of Dr. Brainard in the height of his prosperity.

woman had been greatly shattered, and a miscarriage was constantly apprehended. The doctor was unremitting in his attentions, and finally carried her through her confinement with marked success, exhibiting to the ladies who had taken so much interest in the patient, a fine living child. This was the long desired opportunity, and it did not fail to produce its results. Dr. Brainard immediately became famous. His disinterested sympathy, his goodness of heart, his skillful treatment and his marked success, were now the subject of comment in all circles. At my request, Dr. Goodhue also visited the woman—as I desired to secure his additional testimony in the case—and he too became very favorably impressed with the talents and acquirements of the young practitioner, and extended to him a helping and efficient hand.

"During the winter of 1837–38, Dr. Brainard first communicated to me his project looking to the foundation of Rush College.

"In 1838, a laborer on the canal near Lockport, fractured his thigh, and before union had been completely effected, he came to Chicago on foot, where he found himself unable to walk further and quite destitute. He was taken to the poor-house where he rapidly grew worse, the limb becoming excessively œdematous. A council of physicians was summoned, consisting of Drs. Brainard, Maxwell, Goodhue, Egan, and perhaps one or two others. All were agreed as to the necessity of amputation, but, while Brainard insisted that the operation should be performed at the hip joint, the others urged that removal below the trochanters would answer equally well. The patient was about twenty-three years of age, had an excellent physique, and was, so far as known, of good habits. The operation was assigned to Brainard, and Goodhue was entrusted with the control of the femoral artery, as it emerges from the pelvis. This he was to accomplish with his thumbs; and he had as good thumbs as any man I ever knew. The moment the amputation was effected, Brainard passed one finger into the medullary cavity, and brought out upon it a portion of the medulla which, in the process of disorganization, had become black. As he exhibited it he looked at Goodhue, who simply nodded his head. Not a word was spoken by any one but the patient, and what he said no one knew. Brainard instantly took up the knife and again amputated, this time at the joint, after which the wound was dressed. The double operation occupied but a very short time.

"In about one month the wound had very nearly healed, only a granulating surface of about three-fourths of an inch in length at the upper corner discharged a healthy pus. I was present the last time the wound was dressed, and expected to see the patient speedily discharged as cured. But that night secondary hæmorrhage occurred, a large portion of the wound was opened afresh, and the patient died almost immediately. At the post mortem section, an enormous mass of osseous tubercles was removed from the lungs, liver and heart, and a large, bony neoplasm was found attached to the pelvic bones, and surrounding the femoral artery, so that the mouth of the latter remained patulous. A similar deposit, three inches in diameter, had been found about the fractured femur, and when this was sawn through, the line of demarcation between the neoplasm and the true bone was distinctly discernible.

"The operation was regarded as a success, and it completely established Dr. Brainard's reputation as a surgeon."

There can be but little doubt that a number of amputations at the hip joint must have been performed in this country before the date of the operation thus graphically described by Judge Caton, but it is certain that we have records of only two or three of these at the most. In a recent letter, President J. W. Freer, of Rush College, informs me that the case referred to, was one of enchondroma of the femur, and that the specimen it furnished, adorned the museum of the College until the destruction of the latter by fire.

Some time after Dr. Brainard's arrival in Chicago, he filled the editorial chair of the *Chicago Democrat*, to which the Hon. John Wentworth succeeded.

In the year 1839, Dr. Brainard visited Paris, where he remained for about two years engaged in perfecting himself in the details of professional service, availing himself of the advantages offered in the medical institutions of that city, and laboring with great assiduity. On his return, he delivered a course of medical lectures in St. Louis, and soon after perfected his plans for the establishment and permanent foundation of Rush Medical College. The success which attended the efforts of himself and his associates, not only in this direction but in the publication of the periodical, of which the present MEDICAL JOURNAL AND EXAMINER is the direct and legitimate descendant, is too well known to the profession at large to require comment.

Dr. Brainard revisited Paris in 1852, when he was accompanied by his wife. It was at this time that he obtained permission to prosecute his researches on the subject of poisoned wounds by the aid of experiments upon the reptiles in the Jardin des Plantes. He was then made an honorary member of the Société de Chirurgie of Paris, and of the Medical Society of the Canton of Geneva. In the year 1854, he gained the prize offered by the St. Louis Medical Society for the presentation of his paper on the Treatment of Ununited Fractures—the method he then proposed having since received the endorsement of the entire profession.

A short time before his death he spent a day in Rome, N. Y., with his life-long friend, Mr. Comstock, pleasantly recounting the incidents of his foreign travel, expressing the greatest interest in the prosecution of his work connected with his lectures in the College, and anticipating a return to Europe for a third visit with a view to a still more extended course of investigations.

At the same time he seemed to be impressed with a feeling that he had not much longer to live. In a few weeks from this date, his friend in Rome received the telegraphic announcement of his death. He died of cholera, in the old Sherman House of Chicago, on the 10th day of October, 1866, in the fifty-fifth year of his age.

Dr. Brainard was a master of many of the collateral branches of medical science. He was a botanist and geologist. He excelled also in literature, and his contributions to medical periodicals are many of them masterpieces of terse, vigorous and lucid expression. A generation of men who never looked in his face are yet familiar with his features. He was tall and vigorous in frame, with a large, finely-shaped head, and keen, penetrating eyes. He seemed indeed to possess the three qualities which were considered in the 16th century to be the prerequisites of a good surgeon, viz.: "the eye of a hawk, the hand of a woman, and the heart of a lion." Dr. Brainard's name is graven ineffaceably upon the annals of American Surgery. His successors may well emulate his indomitable perseverance in the face of apparently overwhelming obstacles, his unflagging industry, and the acquisition of the science and skill which perforce spring from these high qualities.

In the Lakeside Annual Directory for 1875-6, is reproduced in *fac simile* the first Directory ever issued in Chicago, dated 1839—the original having been obtained through the courtesy of Henry H. Hurlbut, Esq.

By referring to this, it will be seen that Dr. Brainard's name occurs with those of Drs. S. B. Gray and Betts, as constituting a Board of Health. This Board, it is unnecessary to say, was not organized under any such law as that which provides for the Board of Health as now constituted. Dr. Charles V. Dyer is there registered as City Physician—he had removed to the city three years before, in 1835. Besides these, the Directory contains the names of Dr. Jno. Brinkerhoff, Dr. Clarke, Dr. Levi D. Boone, Dr. Eldridge, Dr. Edmund S. Kimberly, Dr.

Merrick, Dr. Post, and Dr. J. Jay Stuart. Drs. Brinkerhoff, Betts, Post and Stuart, are known to be now dead, besides those whose decease has been heretofore noted in these pages.

Dr. Boone, whose name appears in the list, deserves more than a passing mention. He is the grand nephew of the great Kentucky pioneer, Daniel Boone, and was born on the 8th of December, 1808. He studied medicine in the Transylvania University, came to Illinois in 1829, and, having volunteered as a private in the Black Hawk war, was finally promoted to the Surgeoncy of the 2nd Regiment, 3rd Brigade, Col. Jacob Frye. Dr. Boone came to Chicago in 1836, and still resides here, though he is now gradually withdrawing from the business incidental to the management of his estate.*

The charter for the incorporation of Rush College was obtained from the Legislature in 1837, and was the first instrument issued for a similar purpose to any educational institution in the State of Illinois. The first building occupied by the Faculty was erected in the year 1844, after the designs of Mr. Van Osdel. A passably well-executed cut of this structure was given in the City Directory of the ensuing year.† The names of Professors are thus given: Daniel Brainard, M.D., Professor of Surgery; Austin Flint, M.D., Professor of the Institutes and Practice of Medicine; G. N. Fitch, M.D., Professor of Obstetrics and Diseases of Women and Children; J. V. Z. Blaney, M.D., Professor of Chemistry

* The Directory from which these names have been transcribed was, as might be expected, a very incomplete affair. Mr. Fergus, an early resident of Chicago, has, with considerable labor, compiled a tolerably complete list of the business men of the town in 1839, in which are to be found the following *additional* names, designated as "doctors": Zimon P. Haven, Richard Murphy, William Russell, D. S. Smith, John Mark Smith, Simeon Willard.

† Business Advertiser and General Directory of the City of Chicago, 1845–6. J. W. Norris. This volume is in the valuable collection of Mr. Cooke, of Messrs. Keen, Cooke & Co., publishers of the CHICAGO MEDICAL JOURNAL AND EXAMINER, and I am under obligations to him for the fac simile shown on next page, of the cut of the old Rush College.

and Pharmacy; Jnö. McLean, M.D., Professor of Materia Medica and Therapeutics; and W. B. Herrick, M.D., Professor of Anatomy. Dr. Herrick became subsequently the first President of the Illinois State Medical Society.

THE FIRST RUSH MEDICAL COLLEGE. (1844).

Under the heading of "Physicians and Surgeons" are enrolled twenty-eight names. In addition to three of the professors named above, who were residents of the city, are to be found the names of William Allen, H. H. Beardsly, L. D. Boone, Jno. Brinkerhoff, S. S. Cornell, A. W. Davisson, C. H. Duck, C. V. Dyer, J. W. Eldridge, M. L. Knapp, Philip Maxwell, Aaron Pitney, D. S. Smith, and J. J. Stuart.

In the year 1847, the first general hospital in the city was established, chiefly through the instrumentality of Dr. Brainard and his associates, in a large warehouse on the corner of North Water and Dearborn streets. This was known as "Tippecanoe Hall." It contained one hundred beds, which were well filled, especially during the two succeeding years when ship-fever prevailed, chiefly among the immigrants. Drs. Brainard, Blaney and Herrick constituted the medical staff.

In consequence of the high price of quinine, which was then worth nearly ten dollars per ounce, the county

NEW EDIFICE OF RUSH MEDICAL COLLEGE NOW IN PROCESS OF ERECTION.

authorities who furnished the supplies, refused to provide it for the use of patients, and it was, therefore, found necessary to employ strychnia as a substitute, which answered nearly all purposes in doses of one-eighth of a grain.

Dr. J. W. Freer served as an interne of this institution for two years, and was therefore the first hospital interne in Chicago. In this capacity, he stood first of a long line of industrious and learned successors, who have since distinguished themselves for their attainments in almost every department of medicine.

The first number of the Illinois Medical and Surgical Journal was issued in April, 1844, under the editorial management of James V. Z. Blaney, A.M., M.D. Its reading matter is contained in one form of sixteen pages, just one-sixth the size of the MEDICAL JOURNAL AND EXAMINER, as now published. The very modest introductory sets forth a fair ground for its *raison d'être.* "We have around us three large States: Indiana, Michigan and Illinois—and two extensive territories: Wisconsin and Iowa—filled with medical men of the highest intelligence and most praiseworthy enterprise, and not a single medical journal has been previously issued in all this vast Northwestern region." The number contains an original contribution from Dr. Brainard, on the treatment of false anchylosis by extension, illustrated by a very creditable wood cut; a brief summary of progress in practical medicine, which contains extracts from the 2d Vol. of Pereira's Materia Medica and Therapeutics, the 8th No. of Braithwaite's Retrospect, and the American Journal for January, 1844; and Bibliographical Notices of a Dissector by Erasmus Wilson, and An Anatomical Atlas, by H. H. Smith, M.D.; to both of these reviews Dr. Brainard's initials are appended. There are but two items of general intelligence, both clipped from the Medical News.*

The first meeting with a view to the establishment of the Chicago Medical College, was held in the office of Drs. David Rutter and Ralph N. Isham, on the 12th day of March, 1859.† Drs. Hosmer A. Johnson and Edmund Andrews were then present, together with the gentlemen first named. After a temporary organization had been effected, it was determined to organize a Med-

* This volume is in the possession of Dr. J. Adams Allen, who has been so long identified with the fortunes of this Journal. For a history of the thorny reverses out of which has been plucked its flower of success, consult Dr. Allen's interesting sketch in the January No. for 1874.

† History of the Chicago Medical College—An Introductory Lecture to the College Session of 1870-71. H. A. Johnson, A.M., M.D., Chicago, 1870.

ical Faculty, on the basis of a proposition made by the trustees of the Lind University, and an agreement to that effect was signed, both by the Executive Committee of the University and by the physicians who were there assembled.

The first faculty of the new medical school was constituted as follows: David Rutter, M.D., Emeritus Professor of Obstetrics and Diseases of Women and Children; H. A. Johnson, M.D., Professor of Physiology and Histology; E. Andrews, M.D., Professor of the Principles and Practice of Surgery; R. N. Isham, M.D., Professor of Surgical Anatomy and the Operations of Surgery; N. S. Davis, M.D., Professor of the Principles and Practice of Medicine; W. H. Byford, M.D., Professor of Midwifery and Diseases of Women and Children; J. H. Hollister, M.D., Professor of Physiology and Histology; Dr. Mahla, Professor of Chemistry; M. K. Taylor, M.D., Professor of General Pathology and Public Hygiene; Titus DeVille, M.D., Professor of Descriptive Anatomy; and H. G. Spafford, Esq., Professor of Medical Jurisprudence.

The first course of lectures was given in Lind's Block, on Market, between Randolph and Lake streets, the class consisting of but thirty-three members, of whom nine received, at the commencement exercises, the degree of Doctor of Medicine. In the summer of 1863, arrangements were perfected for the erection of the building on the corner of State and Twenty-second streets, which was occupied by the Chicago Medical College up to the time of its removal, in 1870, to the present elegant and commodious structure on the corner of Prairie avenue and Twenty-sixth street, in close proximity to Mercy Hospital. During the previous year, this institution had become the Medical Department of the Northwestern University.

On the 25th day of April, 1868, the Faculty arranged the curriculum of the College, so that three consecutive courses of lectures should be given, with a separate group of studies for each of the three years of pupilage. The honor which is due the Chicago Medical College for the inauguration of this scheme has been persistently ignored by some of the Medical Schools in the East. It is certainly gratifying to note that this step in the direction of that reform in medical education which is now felt to be imperatively demanded, was first taken in Chicago. It is now a matter of record, and the impartial historian who shall write the history of medicine in the United States, cannot fail to do justice, in this particular, to the young claimant of the West.

THE CHICAGO MEDICAL COLLEGE BUILDING.

The medical board of Mercy Hospital is constituted by the faculty of the adjacent college. The first named institution originated in consequence of a charter obtained from the State legislature, by Dr. John Evans and others, for the establishment of the "Illinois General Hospital of the Lakes." This instrument named Dr. Evans and Judges Dickey and Skinner as Trustees. Nothing, however, had been accomplished toward raising funds or establishing the hospital until the summer of 1850, when Prof. N. S. Davis gave a course of six lectures on the sanitary condition of the city, and the means for its improvement; notice having been previously given th atth proceeds would be devoted to hospital purposes. One hundred dollars were thus realized; and this sum was subsequently increased by the donations of a few private individuals. Twelve beds were at once purchased and placed in the old Lake House Hotel.

The hospital was then opened for the accommodation of patients, nominally under the supervision of the trustees named above, Prof. Davis having charge of the medical, and Prof. Brainard, of the surgical patients. The beds were well filled and supplied the means for daily clinical instruction during the fall and winter of 1850–1. It was placed in charge of the Sisters of Mercy in the spring of 1851, who enlarged its accommodations, and subsequently changed its name to Mercy Hospital. The elegant edifice which they now possess, is capable of accommodating five hundred patients; and it may be added that from the date of the leasing of the old apartments containing twelve beds, to the present—a term of twenty-five years—Prof. N. S. Davis has continuously done service in its wards, as a physician and clinical teacher.

The purpose of this sketch, though but imperfectly fulfilled, has been accomplished, so far as to call attention to the character and circumstances of the early medical practitioners of Chicago. Many of those who immediately succeeded them are still living in our midst, and retain a recollection of events that have transpired in their time, which it would be vain to attempt to record in these pages. I conclude with a brief outline of events connected with the organization of the County Hospital, located in this city, not only because it is at present the largest of our public charities, but also because the recent erection of a new building for its accommodation, seems to mark an era in its history.

During the cholera epidemic of 1854–5, the city authorities established a cholera hospital on the corner of 18th and Arnold streets—the precise location of the building now occupied as a county hospital. The frame buildings then erected were cheaply built, and intended simply to meet immediate necessities. Dr. Brock. McVickar, who was then the City Physician, began at once to urge the Board of Health to erect a permanent city hospital. His importunity caused a movement to take form, which resulted in the erection of the city hospital building, which is at present used for a county hospital.

When completed, in the summer of 1856, the medical staff, as organized by the Board of Health, was constituted of two bodies—the so-called Allopathic and Homœopathic Boards—the former consisting of Drs. Geo. K. Amerman, De Laskie Miller, Jos. P. Ross, Geo. Schlœtzer, Ralph N. Isham, and Wm. Wagner. The members of the regular profession held an indignation meeting soon after, in consequence of the mongrel character of this organization ; and the newly appointed medical staff also held several meetings. Hon. Jno. Wentworth, then Mayor of Chicago, and ex-officio member of the Board of Health, also endeavored to organize a board of reputable practitioners, but failed in the effort.

It then became evident that, the cholera epidemic having subsided, and the city being charged merely with the care of those affected with contagious and infectious diseases, there were no patients for whom the city was obliged to provide! The care of the sick poor, both of the city and county, devolved upon the latter. Thus the building remained unoccupied for a year or two.

In 1858, Drs. Geo. K. Amerman and J. P. Ross associated themselves with four other medical gentlemen, and leased the building from the city authorities, for the purpose of conducting therein a public hospital for the sick. They also secured a contract for the care of the sick poor of the county. The medical board was composed of the gentlemen already named in the first board, with the addition of Drs. Daniel Brainard and S. C. Blake, and the exception of Drs. Isham and Wagner. Clinical instruction was at once given by these gentlemen for eight months in the year, chiefly to the students of Rush College, and continued till the summer of 1863.

At that date the hospital was taken by the Government authorities—Chicago having been made a military post during the War of the Rebellion, and Drs. Ross and Amerman were placed in charge of the hospital on contract service, under the control of the surgeon of the post, Dr. Brock. McVickar. In the course of a few months, the institution was changed into a Government Hospital for the Eye and Ear, and placed in charge of Dr. Jos. Hildreth, in whose care it remained till the close of the war. It was then named the DeMarr Eye and Ear Hospital.

Drs. Ross and Amerman at once actively interested themselves in the re-establishment of the hospital. On looking over the field, they became convinced not only that the county authorities would look with favor upon the organization of a county hospital, but also that, in order to compass the end, it would be necessary for one of them to become a politician. Dr. Amerman accordingly secured his election as a Supervisor, and, in 1866, the first year of his service as such, he inaugurated and organized the Cook County Hospital, for the care of the indigent poor, and for the clinical instruction of medical students. During this same year, Dr. Amerman was obliged to relinquish his official position, on account of ill health, and Dr. J. P. Ross was at once elected to fill the vacancy, as Supervisor and Chairman of the Hospital Committee. The duties incident to this position he continued to discharge for the two succeeding years.

All this was undertaken for the sole purpose of permanently establishing and perpetuating the institution. It is therefore evident that to Dr. J. P. Ross and his old friend and colleague, Dr. G. K. Amerman, is solely due the honor of conducting to a successful issue, the plans for the development of this great municipal charity.

The names of other public institutions and charities of Chicago, in which the profession of the city is interested, together with the date of the establishment of each, are appended in a note.*

The medical profession of Chicago enters upon this centennial year of national existence, with the names of three hundred and sixty-six physicians and surgeons enrolled upon its register. Many of these are both honorable and honored. Of the record made in the past they need not be ashamed; in much that has been accomplished they feel a just pride.

At the same time the experiences of the last forty years have taught them the sources of their weakness and therefore of their danger. If they have learned anything it is this, that to be conscious of deficiency and danger is to acquire the alphabet of knowledge—that to render any body of men a living power in a community, it is needful that each individual member of it should exert a wise, wholesome and weighty influence in the circle where he moves. They look, therefore, rather to their inherent capabilities than to any legislative or other source, for growth in reputation and authority. Already a tendency has been developed, for the crystallization of this power and authority, about certain defined centres. That this process is destined to continue until its standards are elevated, its code admired and respected, and its

* Chicago Medical Society, 1836; Chicago Protestant Orphan Asylum, 1849; Mercy Hospital, 1850; Illinois State Medical Society, 1850; Saint Joseph's Orphan Asylum, 1849; Chicago Academy of Sciences, 1857; House of the Good Shepherd, 1859; Home for the Friendless, 1859; Illinois Charitable Eye and Ear Infirmary, 1858; Chicago College of Pharmacy, 1859; Chicago Relief and Aid Society, 1857; Nursery and Half Orphan Asylum, 1860; St. George's Benevolent Society, 1860; St. Luke's Hospital, 1863; Old People's Home, 1865; Erring Woman's Refuge, 1865; Chicago Hospital for Women and Children, 1865; Alexian Brothers' Hospital, 1860; Central Dispensary, 1867; St. Joseph's Hospital, 1869; Washingtonian Home, 1867; Uhlich Evangelical Lutheran Association, 1869; State Microscopical Society, 1869; Woman's Hospital Medical College, 1870; Woman's Hospital State of Illinois, 1871; Cook County Department of Public Charities, 1872; Foundlings' Home, 1871; Chicago Society of Physicians and Surgeons, 1872; Chicago Medico-Historical Society, 1874; Chicago Medical Press Association, 1874; Orphan Girls' Home, 1874.

accidental excrescences removed, no one can doubt. Then and only then will it become as fair and as forcible in the view of the public, as in the vision of its most ardent representatives.

## Figures and Tables

**Figure f1:**
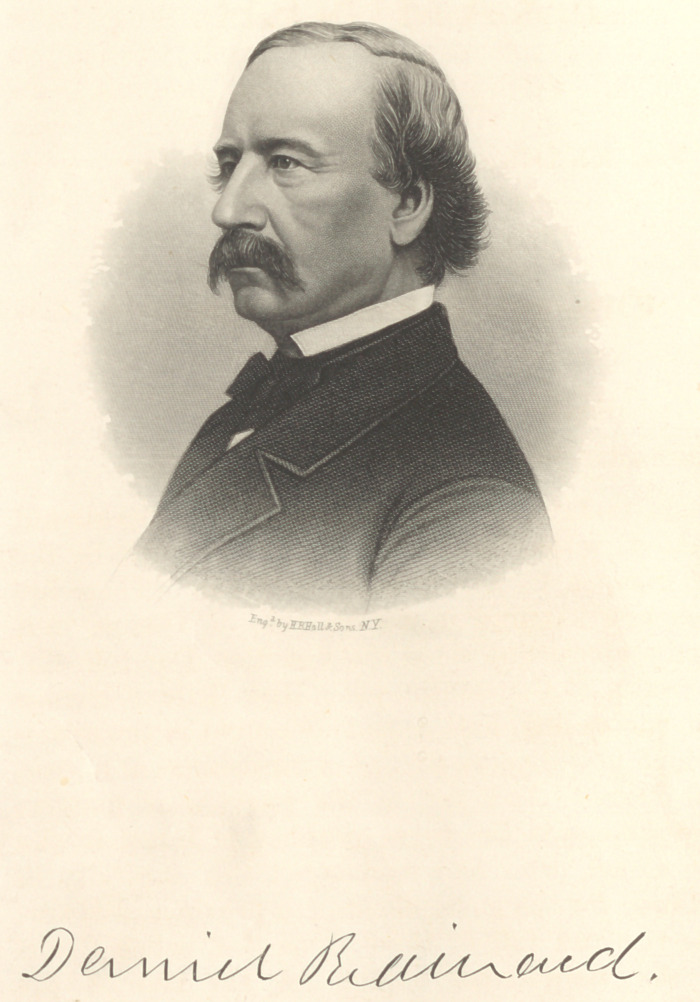


**Figure f2:**
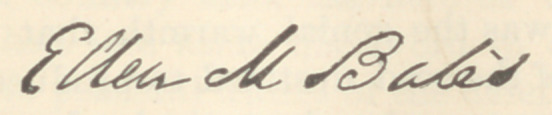


**Figure f3:**
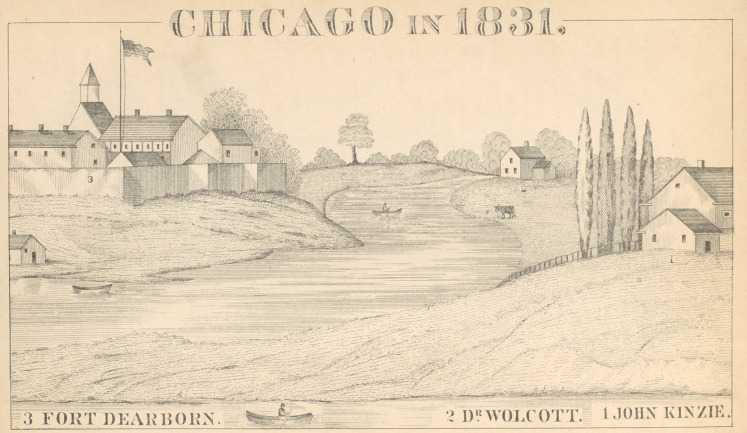


**Figure f4:**
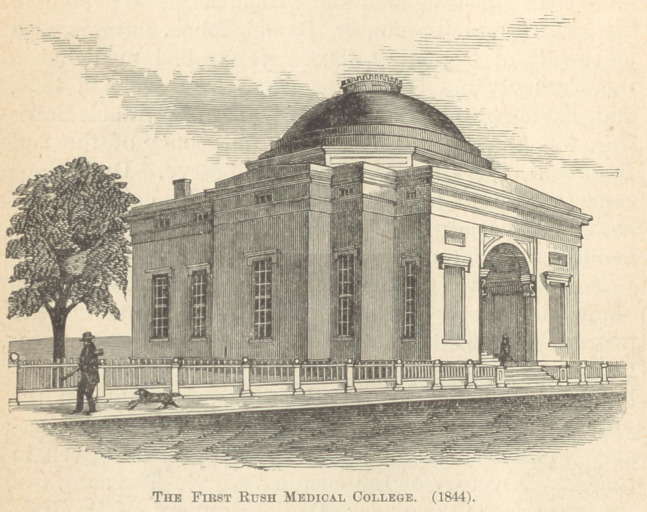


**Figure f5:**
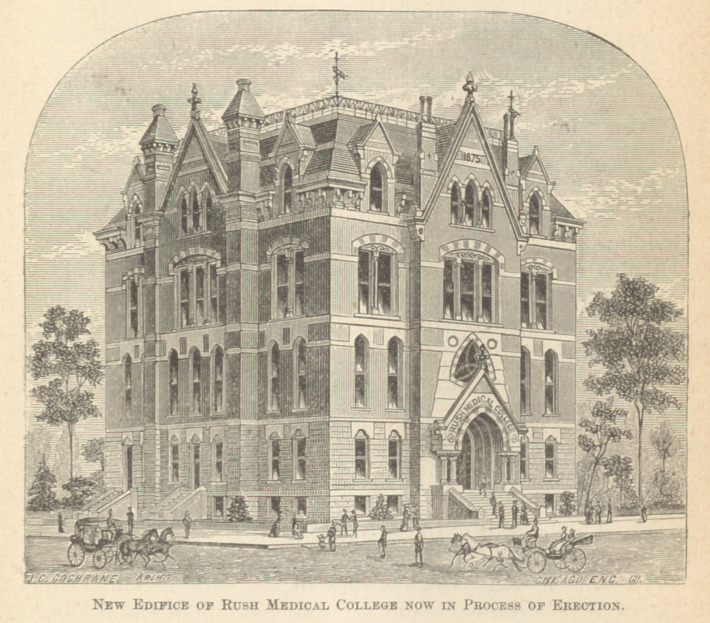


**Figure f6:**